# Clinical Utility and Limitations of Intraoperative Monitoring of Visual Evoked Potentials

**DOI:** 10.1371/journal.pone.0120525

**Published:** 2015-03-24

**Authors:** Yeda Luo, Luca Regli, Oliver Bozinov, Johannes Sarnthein

**Affiliations:** 1 Neurosurgery Department, University Hospital Zurich, Zurich, Switzerland; 2 Zurich Neuroscience Center, Eidgenössische Technische Hochschule Zurich, Zurich, Switzerland; University of Western Australia, AUSTRALIA

## Abstract

**Objectives:**

During surgeries that put the visual pathway at risk of injury, continuous monitoring of the visual function is desirable. However, the intraoperative monitoring of the visual evoked potential (VEP) is not yet widely used. We evaluate here the clinical utility of intraoperative VEP monitoring.

**Methods:**

We analyzed retrospectively 46 consecutive surgeries in 2011-2013. High luminance stimulating devices delivered flash stimuli on the closed eyelid during intravenous anesthesia. We monitored VEP features N75 and P100 and took patients' preoperative and postoperative visual function from patient charts. Postoperative ophthalmologic workup was performed in 25 (54%) patients and preoperatively in 28 (61%) patients.

**Results:**

VEP recordings were feasible in 62 of 85 eyes (73%) in 46 patients. All 23 eyes without VEP had impaired vision. During surgery, VEPs remained stable throughout surgery in 50 eyes. In 44 of these, visual function did not deteriorate and three patients (6 eyes) developed hemianopia. VEP decreased transiently in 10 eyes and visual function of all was preserved. VEPs were lost permanently in 2 eyes in two patients without new postoperative visual impairment.

**Conclusions:**

Satisfactory intraoperative VEP monitoring was feasible in all patients except in those with severe visual impairment. Preservation of VEPs predicted preserved visual function. During resection of lesions in the visual cortex, VEP monitoring could not detect new major visual field defects due to injury in the posterior visual pathway. Intraoperative VEPs were sensitive enough to detect vascular damage during aneurysm clipping and mechanical manipulation of the anterior visual pathway in an early reversible stage. Intraoperative VEP monitoring influenced surgical decisions in selected patients and proved to be a useful supplement to the toolbox of intraoperative neurophysiological monitoring.

## Introduction

Neurosurgery near the visual pathway carries a significant risk of visual impairment. In high risk patients with lesions such as pituitary adenoma, craniopharyngioma or aneurysms, intraoperative monitoring for preservation of the visual function is desirable. However, the intraoperative monitoring of visual evoked potentials (VEPs) is not yet widely used and its usefulness is debated in conflicting reports in the literature. First reports [1–3] described specific VEP features which were monitored intraoperatively to improve the visual outcome. Further studies [4–9], however, were more critical and considered intraoperative VEP to be unstable and variable. Technical difficulties interfered with the feasibility. The correlation between VEP results and visual outcome were disappointing. Sporadic favorable [10] reports were encountered by contrary findings [11,12].

Only recently, high rates of feasibility were reported in a number of publications and stable VEPs were associated with good postoperative visual function [13–16]. While evoked potential recording and anesthesia protocols have remained unchanged, possibly the introduction of high-luminance devices with supramaximal stimulation contributed to the success of VEP by improving the constant stimulus delivery. We therefore introduced high-luminance VEP stimulating devices in our clinic. In the current study we evaluate the feasibility and utility of the intraoperative monitoring of the VEP.

## Methods

### Patients

We selected all consecutive patients who underwent cranial or transsphenoidal surgical procedures where intraoperative VEP was monitored in our institution from November 2011 to December 2013. With this criterion we included 46 patients (mean age 50 y, range 2–81 y, 28 female, Table 1). We recorded bilateral VEP in all but some cases with unilateral indication, resulting in VEP of 85 eyes. There were 42 patients with intracranial tumors, three with aneurysms and one with an arachnoid cyst. All patients underwent pre- and postoperative neurological examination of visual function. Ophthalmological documentation including Goldmann kinetic perimetry was performed in 28 of 46 (61%) patients preoperatively and in 25 of 46 (54%) patients postoperatively.

**Table 1:** Patient characteristics.

Case No.	Age (yrs), Sex	Pathology(location)	Associated visual disturbance	Number of eyes	VEP feasible	Preoperative visual function	Postoperative visual function	
1	19, M	medulloblastoma metastasis (temporal and occipital lobe)	homonymous hemianopia	2	yes	normal	unchanged	TN
2	12, F	pilocytic astrocytoma (right optic nerve)	unilateral field loss	2	yes	visus impairment (rt)	unchanged	TN
3	45, F	oligodendroglioma (parietal lobe)	homonymous hemianopia	2	yes	normal	homonymous hemianopia	FN
4	67, M	glioma (temporal and occipital lobe)	homonymous hemianopia	2	yes	normal	unchanged	TN
5	50, F	meningioma (optic chiasma)	bitemporal hemianopia	2	yes	normal	unchanged	TN
6	59, F	pituitary adenoma (optic chiasma)	bitemporal hemianopia	2	yes	normal	unchanged	TN
7	54, F	haemangiopericytom (right optic nerve)	unilateral field loss	2	yes	normal	unchanged	TN
8	50, F	glioblastoma (temporal and occipital lobe)	homonymous hemianopia	2	yes	homonymous quadrantanopsia	homonymous hemianopia	FN
9	30,M	pituitary adenoma (optic chiasma)	bitemporal hemianopia	2	yes	normal	unchanged	TN
10	50, M	chondrosarcoma (left optic nerve)	unilateral field loss	1	yes	normal	unchanged	TN
11	54, F	meningioma (right optic nerve)	unilateral field loss	2	yes	visus impairment (rt)	unchanged	TN
12	62, F	meningioma (left optic nerve)	unilateral field loss	2	yes	normal	unchanged	TN
13	19, M	pituitary adenoma (optic chiasm)	bitemporal hemianopia	2	yes	normal	unchanged	TN
14	68, F	meningioma (left optic nerve)	unilateral field loss	2	yes	normal	unchanged	TN
15	60, F	glioblastoma (parietal and occipital lobe)	homonymous hemianopia	2	yes	normal	homonymous hemianopia	FN
16	25, M	pituitary adenoma (left optic nerve, optic chiasma)	bitemporal hemianopia	2	yes	visus impairment(lt)	unchanged	TN
17	75, M	pituitary adenoma (optic chiasma)	bitemporal hemianopia	2	yes	visus impairment (rt) and temporal visual field defect (rt)	improved	TN
18	59, F	meningioma (right optic tract)	homonymous hemianopia	2	yes	homonymous quadrantanopsia	unchanged	TN
19	14, F	pituitary adenoma (optic chiasma)	bitemporal hemianopia	1	yes	homonymous hemianopia	unchanged	TN
20	59, M	metastasis (temporal and occipital lobe)	homonymous hemianopia	2	yes	normal	unchanged	TN
21	49, F	meningioma (optic chiasma)	bitemporal hemianopia	2	yes	visus impairment and bitemporal hemianopia	unchanged	TN
22	58, F	meningioma (left optic nerve)	unilateral field loss	2	yes	normal	unchanged	TN
23	38, F	craniopharyngioma (left optic nerve, optic chiasma)	unilateral field loss, bitemporal hemianopia	2	yes	visus impairment (lt) and bitemporal hemianopia	unchanged	TN
24	57, M	arachnoid cyst (left optic nerve, optic chiasma)	unilateral field loss, bitemporal hemianopia	2	yes	visus impairment (lt) and heteronymous hemianopia	unchanged	TN
25	34, M	haemangioma cavernosum (occipital lobe)	homonymous hemianopia	2	yes	normal	unchanged	TN
26	63, M	pituitary adenoma (optic chiasma)	bitemporal hemianopia	2	yes	bitemporal hemianopia	complete remission	TN
27	39, F	craniopharyngioma (optic chiasma)	bitemporal hemianopia	1	yes	temporal scotoma	unchanged	TN
28	47, F	aneurysm (occipital lobe)	homonymous hemianopia	1	yes	normal	unchanged	TN
29	55, F	pituitary adenoma (optic chiasma)	bitemporal hemianopia	2	yes	temporal hemianopia (lt)	unchanged	TN
30	35, F	pilocytic astrocytoma (right optic tract)	homonymous hemianopia	2	yes	homonymous hemianopia	improved	TN
31	65, F	chondrosarcoma (left optic nerve)	unilateral field loss	2	yes	left visus impairment	unchanged	TN
32	32, F	meningioma (left optic nerve)	unilateral field loss	2	yes	right visus impairment	unchanged	FP
33	36, M	meningioma (left optic nerve)	unilateral field loss	2	yes	quadrantanopsia (lt)	unchanged	TN
34	79, F	meningioma (left optic nerve)	unilateral field loss	2	yes	visus impairment (lt)	unchanged	TN
35	58, F	aneurysm (right hemisphere)	unilateral field loss, hemianopia	1	yes	visus impairment (rt)	unchanged	FP
36	49, M	pituitary adenoma (optic chiasma, left optic tract)	bitemporal and homonymous hemianopia	2	yes	homonymous hemianopia	unchanged	TN
37	81, F	pituitary adenoma (optic chiasma)	bitemporal hemianopia	2	yes	visus impairment	unchanged	TN
38	73, F	B-Cell lymphoma (parietal and occipital lobe)	homonymous hemianopia	2	yes	homonymous hemianopia	improved	TN
39	37, M	pituitary adenoma (optic chiasma)	bitemporal hemianopia	2	no	bitemporal hemianopia	improved	
40	38, F	craniopharyngioma (optic chiasma)	bitemporal hemianopia	2	no	visus impairment and bitemporal hemianopia	improved	
41	13, F	astrocytoma (right optic nerve, optic chiasma)	unilateral field loss, bitemporal hemianopia	2	no	visus impairment (rt) and bitemporal hemianopia	unchanged	
42	52, M	pituitary adenoma (optic chiasma)	bitemporal hemianopia	2	no	visus impairment and bitemporal hemianopia	improved	
43	76, F	chordoma (optic chiasma)	bitemporal hemianopia	2	no	visus impairment (lt) and bitemporal hemianopia	unchanged	
44	2, M	pilomyxoid astrocytoma (optic chiasma)	bitemporal hemianopia	1	no	visus impairment	n/a	
45	56, M	meningioma (right optic nerve)	unilateral field loss	1	no	amaurosis (lt), visual field defect (rt)	unchanged	
46	17, M	aneurysm (left hemisphere)	unilateral field loss, hemianopia	2	no	visus impairment and quadratanopsia	Hemianopia	

### Ethics statement

Retrospective collection of personal patient data and scientific workup was approved by the institutional ethics review board (Kantonale Ethikkommission KEK-ZH 2012–0212). The data were collected retrospectively. Our institutional review board waived the need for written informed consent from the participants (KEK-ZH 2012 0212, Kantonale Ethikkommission Zurich). Patient information was anonymized and de-identified prior to analysis.

### Anesthesia management

Following the standard protocol for neurosurgical interventions, anesthesia was induced with intravenous application of the sedative drug Propofol (4 to 8 mg/kg/min), the opioid analgesic Remifentanil (1–2 μg/kg/min) and the skeletal muscle relaxant Atracurium (0.5 mg/kg). If VEPs were monitored concurrently with other evoked potentials, Atracurium was omitted after intubation because of its interference with electrophysiological monitoring and mapping of motor function.

### VEP stimulation device and parameters

**Fig 1:**
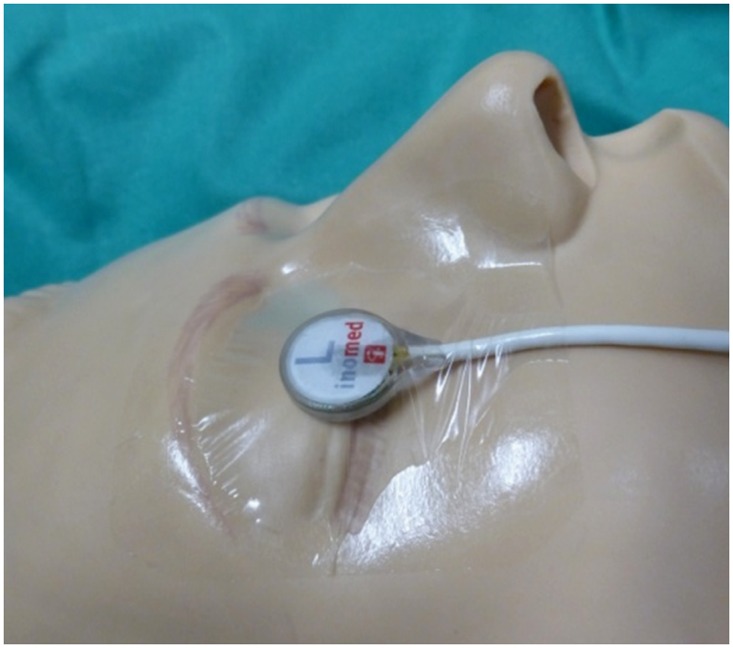
Light-stimulating device. Each device contains 19 red (654 nm) diodes that provide illuminance up to 25000 Lux.

After the induction of anesthesia, transparent eye patches were placed on the closed eyes. Then the light-stimulating device was placed on the eyelids and covered with another transparent eye patch. For the first 36 patients, we used devices from Unique Medical Co. Ltd, Tokyo [16] with pulse length 40 ms. For the subsequent 10 Patients, we used stimulation devices from Inomed (Inomed Medizintechnik GmbH, Germany, www.inomed.com, Fig. 1) with pulse length 10 ms, which were also tested for safety in intraoperative 3 Tesla MRI [17]. The stimulus frequency (1.1 Hz) was chosen to reduce line hum in the VEP. The red light-emitting diodes provided (654 nm) illuminance up to 26000 Lux. While we set the illuminance to 20000 Lux in our first surgeries ensure feasibility of the VEP, we also obtained reliable results with 1000 Lux in some patients.

### Recording electrode sites

Needle electrodes were inserted subcutaneously according to the international EEG 10–20-system. The electrodes were placed on the bilateral preauricular point (A1, A2), the left and right occiput (O1, O2), the occipital midline (Oz) and along the cortical midline (Cz, Fz). A1 and A2 were linked (A+) and served as recording reference.

### VEP data acquisition and analysis

Flash VEP was recorded with the Inomed ISIS System. Recording was performed in a montage of nine channels with the sites O1, O2, Oz against Fz, Cz and linked A1 and A2 (A+). We averaged 100–200 flash stimulations to obtain one VEP waveform.

VEPs were band-passed at 5–100 Hz, displayed in a 500 ms-window with 10 μV/Division and negative up. N75 and P100 peaks were selected in the latency range 50–150 ms after the end of the stimulation pulse. The N75 amplitude was taken as the voltage difference between the peaks N75 and P100. The P100 amplitude was taken as the difference between P100 and the following negativity.

VEP monitoring was performed after completion of patient setup. At least two responses were recorded to confirm the reproducibility of the VEP waveform, preferably before surgical manipulation. When the surgical manipulation approached the area of interest, a baseline was set as the control level for further monitoring.

### Warning criterion

We applied a two-step warning criterion to take into account the individual spontaneous variability of VEP responses. First, a N75 amplitude decrease to less than 50% of the baseline was taken as the warning criterion. Whenever VEP amplitude decreased below the warning criterion, first technical failures were excluded and then anesthesia parameters were checked. If the VEP change beyond the warning criterion could not be explained by a technical issue or the anesthesia regimen, a temporary warning was issued to the surgeon. In a second step, the succeeding VEP was used to either confirm or reject the warning. If the warning criterion was not reached, VEPs were referred to as “stable”.

### Statistical analysis

Over the patient group, amplitude and latency of the N75 and P100 peaks were compared between eyes with preoperative intact visual function and eyes with preoperative visual impairment.

Statistical analyses were performed with IBM SPSS Statistics 22 (www.ibm.com) and custom scripts in Matlab R2010a (www.Mathworks.com). For ratios, the 95% confidence intervals (CI) were obtained on the basis of the binomial distribution. Distributions were compared by non-parametric testing. Statistical significance was established as p<0.05.

We examined the correlation between intraoperative VEP features and postoperative visual function. The outcomes of VEP and neurological examinations were dichotomized for statistical treatment in contingency tables with the chi² test. A contingency table contains the elements true positive (TP), true negative (TN), false positive (FP) and false negative (FN). Derivations of these are the sensitivity or true positive rate TPR = TP/(TP+FN), the false positive rate FPR = FP/(FP+TN), the accuracy ACC = (TP+TN)/(TP+TN+FP+FN)), the specificity 1-FPR, the negative predictive value NPV = TN/(TN+FN), and the positive predictive value PPV = TP/(TP+FP).

## Results

### Feasibility of intraoperative VEP

Intraoperative monitoring of VEP recordings was feasible in 62 of 85 eyes (73%, CI [62–82%]) and in 38 of 46 patients (83%, CI [69–92%]). There were no complications attributable to VEP recording. Intraoperative VEP monitoring was not feasible in 23 eyes (17 patients). In 8 patients listed in Table 1, VEP could not be monitored from either eye. In the other 9 patients, VEPs were monitored bilaterally but were only feasible in the eye with intact vision. The preoperative visual function of all these 23 eyes was impaired (12 eyes had a combination of visual acuity impairment and visual field defect; 7 eyes had reduced visual acuity; 4 eyes had an impairment of the visual field alone).

Recordings from Oz in the montage with reference A+ yielded the highest success rate (53/74, 72%, Table 2). In general, the recordings with reference A+ provided the highest rate of feasible VEP recordings (133/220, 60%).

**Table 2:** Utility of VEP recording channels.

Channel	Number of attempts	Number of Success	Success rate [%]
O1/A+	73	42	58
**Oz/A+**	**74**	**53**	**72**
O2/A+	73	38	52
O1/Cz	63	31	49
Oz/Cz	61	40	66
O2/Cz	61	31	51
O1/Fz	33	16	48
Oz/Fz	49	26	53
O2/Fz	51	15	29

### Correlation of intraoperative VEP findings and postoperative visual function

Correlation of VEP amplitude and visual outcome are shown in Tables 3 and 4. In 50 eyes with stable VEP, visual function remained unchanged in 36 eyes, improved in 8 eyes, and 3 patients (6 eyes) developed homonymous hemianopia. VEP decreased transiently in 10 eyes and visual function of all these was preserved. VEPs were lost permanently in 2 eyes in two different patients without new postoperative visual impairment.

**Table 3:** Relationship between VEP amplitude and visual outcome.

Intraoperative VEP amplitude		Visual outcome	
Unchanged	50	Unchanged	36
		Improved	8
		Visual field defect	6
Transient loss	10	Unchanged	10
Permanent loss	2	Unchanged	2

VEP was feasible in N = 62 eyes.

**Table 4:** Contingency table of VEP and new visual impairment.

	Impaired visual function	Preserved visual function	Total
Permanent VEP loss	0 (TP)	2 (FP)	2
Unchanged VEP	6 (FN)	54 (TN)	60
Total	6	56	62

Three patients (6 eyes, patients 3, 8 and 15) showed homonymous hemianopia at the postoperative neurological examination, which was confirmed ophthalmologically in two patients. The intraoperative VEP in all patients had never reached the warning criterion (false-negative FN).

In two eyes of two patients (patient 32 and 35), the postoperative neurological and ophthalmological examination revealed no new visual impairment. However, in these patients the initial reproducible VEPs were lost permanently during surgery (false-positive FP) for unknown reasons. Since the dura was still closed at the time of VEP loss, no direct surgical maneuver could have caused damage to the visual pathway and the VEP loss did not influence surgical decisions.

### Distribution of VEP latency and amplitude

**Fig 2:**
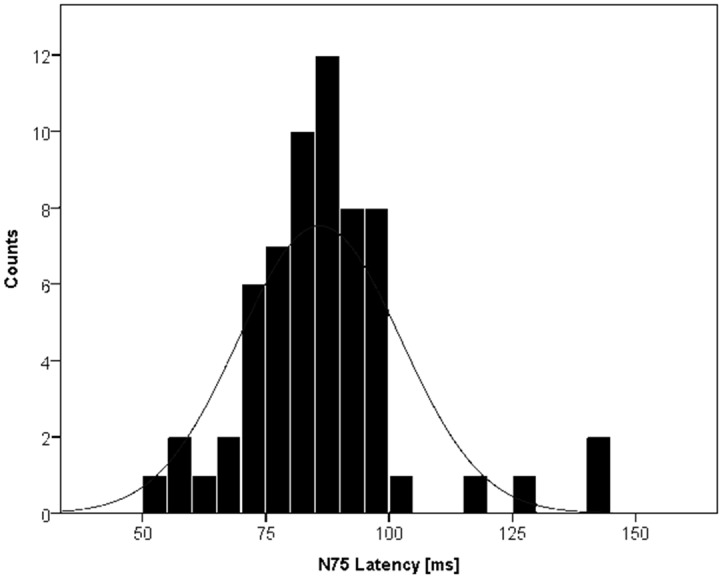
Distribution of N75 latencies. The median N75 latency was 87 ms.

The distribution of N75 latencies in all 62 eyes, where VEP was feasible, (Fig. 2) had a median of 87 ms (range 51–142 ms). The spread may be due to effects of anesthesia. The median N75 amplitude was 2.8 μV (0.7–19.4 μV). The median amplitude and latency of P100 was 2.2 μV (0.2–14.1 μV) and 106 ms (65–161 ms).

**Fig 3:**
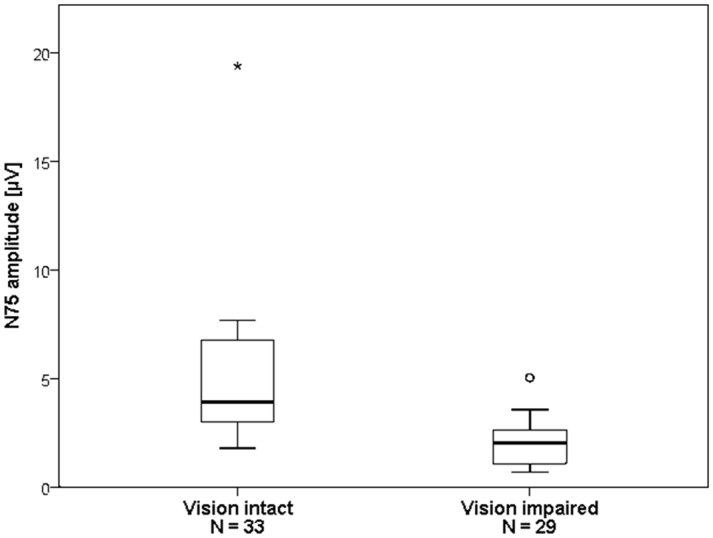
Impaired vision is associated with reduced N75 amplitude. Box plot showing the distribution of the N75 amplitude in eyes with intact and impaired preoperative visual function. The median N75 amplitude for the intact vision group (3.92±4.09 μV, range 1.78 to 19.39 μV) was higher than that of the impaired vision group (2.03±1.16 μV, range 0.68 to 5.03 μV). Mann-Whitney *P* < 0.001.

We divided the 62 eyes, where VEP was feasible, into the groups ‘vision intact’ (*n*_1_ = 33) and ‘vision impaired’ (*n*_2_ = 29) based on their preoperative visual function (Fig. 3). Between the groups there was no statistically significant difference between latencies (N75 *P* = 0.61; P100 *P* = 0.53). In the ‘vision intact’ group, amplitudes were significantly higher both for N75 (*P* < 0.001) and P100 (*P* < 0.001). VEPs with a large N75 amplitude (3. Quartile) were associated with a high stability of the VEP waveform over the course of surgery (Fisher’s exact test P < 0.02).

### False-positive transient VEP loss

In patient 27, shortly after the osteotomy, a generalized epileptic seizure occurred. During the seizure, the VEP was lost. The seizure was immediately intercepted with sodium thiopental. The swelling of the brain was controlled by Mannitol. After the seizure, the VEP recovered to its original waveform.

In patient 5 and 12, VEP was lost during the drilling of burr holes and bone milling. In both patients, the VEPs recovered after the manipulation.

In patient 11 and 14, VEP waveform disappeared for 20 min after application of 300 mg sodium thiopental and for 20 min after application of 5 mg of Dormicum, respectively.

In patient 33, VEP disappeared transiently and neither technical/pharmaceutical issues nor a surgical maneuver could be identified as a cause. The VEP recovered after 30 min and remained reproducible until the end of surgery.

### Representative cases

**Fig 4:**
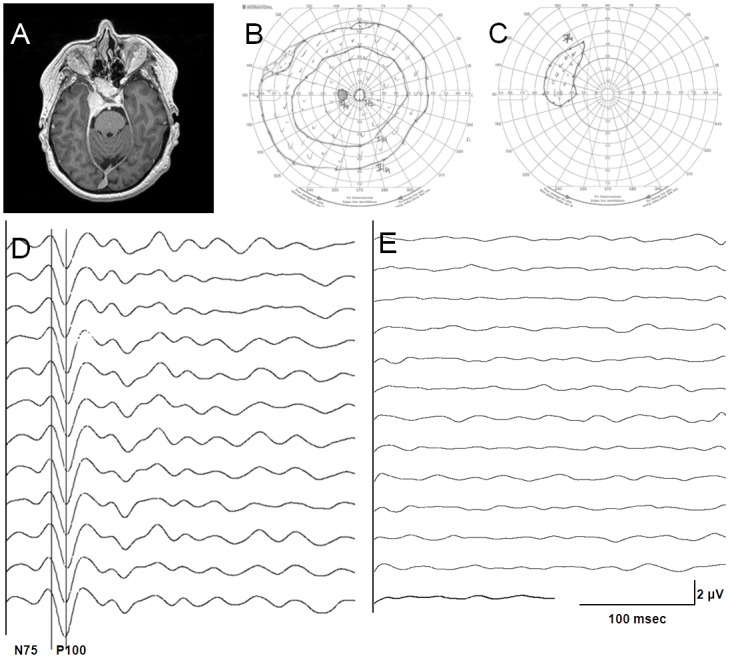
Asymmetric vision and VEP in a patient with a lesion in the anterior visual pathway. **(A)** Anterior skull base meningioma infiltrating the sinus cavernosus of the sphenoid sinus and the right optic canal (patient 11). **(B)** Goldmann perimetry for the left eye: intact visual field. **(C)** Goldmann perimetry for the right eye: major visual field defect. **(D)** VEP of the left eye was highly reproducible throughout surgery (N75 at 74 ms, P100 at 83 ms). **(E)** In the right eye, the patient’s vision was reduced eye and VEP recording was not feasible.

This 54-year-old woman (Patient 11) had a sphenopetroclival meningo-theliomatous meningioma (WHO Grade I) causing a compressive optic neuropathy on her right side (Fig. 4). The preoperative ophthalmological examination of her right eye showed severely impaired visual acuity and a major visual field defect, only leaving a small upper nasal quadrant intact. Her left eye showed normal visual function. The endoscopic approach was adopted for the transsphenoidal tumor resection. The left VEP was stable throughout surgery while the right VEP was flat. At the postoperative neurological examination, the patient showed no new visual impairment.

**Fig 5:**
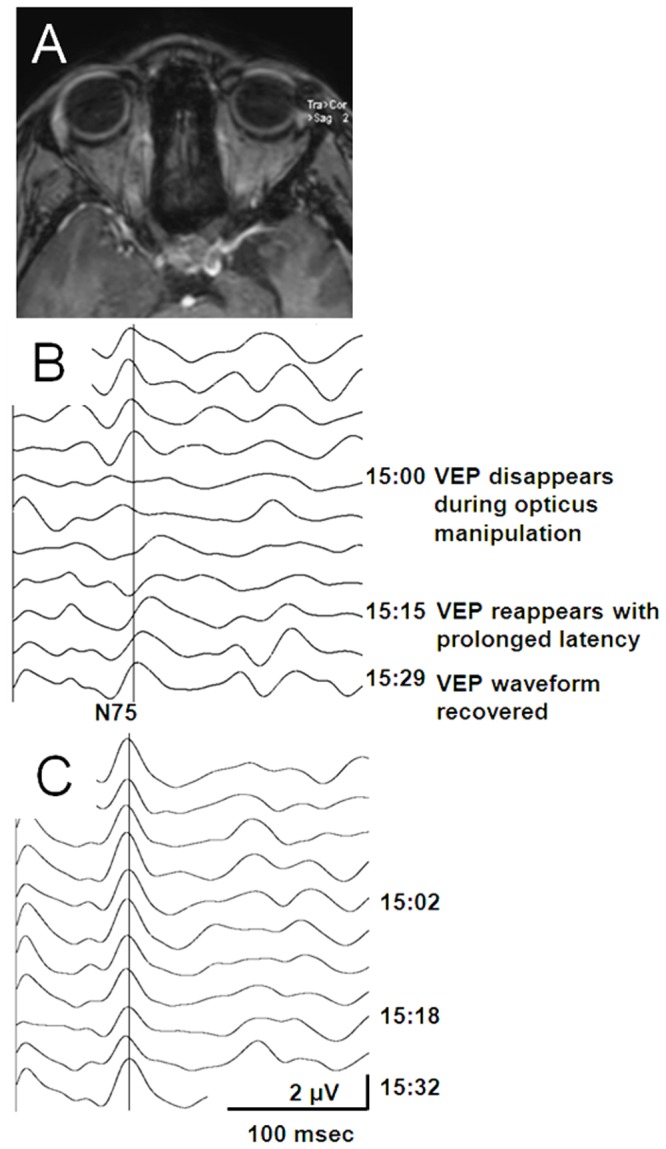
Transient VEP loss due to manipulation of the optic nerve. **(A)** Preoperative MR image showing a recurrent craniopharyngeoma compressing the optic nerve. **(B)** Initially, VEP responses of the left eye were reproducible (N75 at 80 ms) in channel Oz/A+. Later in surgery the surgeon manipulated the left optic nerve. At 15:00, the VEP decrease reached the warning criterion (50%) and a warning was issued to the surgeon who consequently altered the surgical strategy. At 15:15, VEP recovered about 15min. later with prolonged latencies. At 15:29, the VEP waveform recovered at the end of the procedure. **(C)** VEP responses of the right eye in channel Oz/A+ remained unchanged during the manipulation.

The MR image of a 38-year-old woman (Patient 23, Fig. 5) presented a recurrent craniopharyngioma, which progressed in size. The preoperative neurological examination showed a heteronymous bitemporal hemianopia, accentuated on the left side. The left visual acuity allowed for finger counting only. The endoscopic resection was conducted through a transnasal approach with a transseptal flap. At the beginning of the surgical procedure, we confirmed the feasibility of VEP monitoring for both eyes separately. During tumor dissection near the optic apparatus, the left optic nerve was stretched and the left eye VEP decreased to less than 50% of the baseline. A warning was issued to the surgeon who consequently altered the surgical strategy. The VEP recovered about 15 min after the warning.

**Fig 6:**
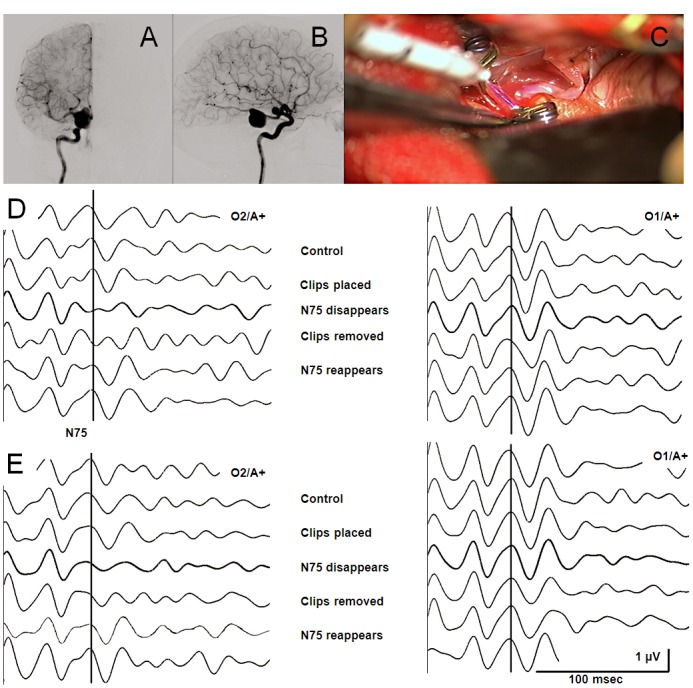
Transient VEP loss due to aneurysm clipping. **(A)** Sagittal view. Fusiform dissecting aneurysm (23mmx20mmx22mm) of the posterior cerebral artery in the P2 segment (Patient 23). Clinically, it manifested with a light hemiparesis on the left side. **(B)** Lateral view. **(C)** Intraoperative screenshot after the placement of two clips. **(D)** Initially, VEP responses were reproducible (N75 at 93 ms) in channel O2/A+. The first clip placement led to a VEP loss after three minutes. Two minutes after the occlusion was released, the VEP recovered to its original waveform. The VEPs in channel O1/A+ remained unchanged during clipping. **(E)** A second transient clip placement resulted in the same pattern of VEP change.

A-47-year-old patient (Patient 28) had a dissecting aneurysm in the right P2 segment with diameter 20 mm (Fig. 6). A temporo-basal approach from the right side was conducted. The first clip placement led to a VEP loss after three minutes. Two minutes after the occlusion was released, the VEP recovered to its original waveform. A second transient occlusion resulted in the same pattern of VEP loss and recovery. Subsequently, the VEP remained reproducible until the end of the surgery.

## Discussion

### Feasibility of intraoperative VEP monitoring depends on preoperative visual function

In our study, we obtained reproducible VEP in all eyes with intact preoperative visual function. In eyes with impaired visual function, VEP was obtained in only 29 of 52 (56%) of cases. In impaired eyes, the N75 amplitude was significantly lower than in eyes with intact vision. We were not able to characterize a minimum visual function to predict feasibility of intraoperative VEP. Detailed ophthalmological documentation preoperatively might help to find optimal criteria for patient selection. This finding is in line with other studies that indicate that VEP recordings can only be obtained in patients without severe visual impairments [13, 16, 18, 19]. We did not encounter any complications with our new device for VEP recording and therefore consider its use to be safe.

### Surgical maneuvers result in immediate VEP loss

The aim of intraoperative VEP monitoring is to alert the surgeon to consequences of surgical maneuvers. In our series, there were 3 instances, where a surgical maneuver could be related to immediate VEP loss. In one surgery (case 23), the optic nerve was manipulated and in another surgery (case 28) the PCA was clipped twice. In all these instances the surgeon was alerted and subsequently changed the surgical strategy. The VEP recovered within a few minutes (Figs. 5 and 6).There were no new postoperative deficits. In these cases, intraoperative VEP monitoring provided early detection of damage when it was still in a reversible stage and has influenced the surgical strategy. Similar direct correlation has also been described in other studies including VEP changes during extraaxial manipulation [10, 18] and arterial clipping [16, 19].

### Susceptibility of VEP to interfering factors

Anesthesia regiment is known to have strong effects on intraoperative VEP monitoring [4, 10, 20–23] with a main susceptibility to volatile agents. Spontaneous fluctuation of VEP monitoring with TIVA is lower [13–16, 18]. Nevertheless, changes in anesthesia regimen with sodium thiopental (patient 14) and dormicum (patient 11) interfered with VEP monitoring in our study.

Additionally, there were cases of transient VEP loss associated with a generalized epileptic seizure (patient 27), and to bone drilling (patients 5 and 12). In these cases the reason for the loss was well identifiable. In case 33, the origin remained unknown. In all cases of transient VEP loss, the visual function remained unchanged. In two eyes of patient 32 and 35 with permanent VEP loss, VEPs were feasible at the beginning of the surgery but were lost for unknown reasons before dura opening. Pharmaceutical and technical issues were excluded. Since the dura was still closed, no direct surgical maneuver could have caused damage to the visual pathway. The loss of VEPs did not influence surgical decisions. During the course of surgery, the waveform did not recover to above the warning criterion. Even though we could not identify a specific cause for the VEP loss, the point in time of the loss leads us not to expect postoperative visual deterioration.

### Association between intraoperative VEP and postoperative visual function

Analysis of the contingency table (Table 3) resulted in a specificity of 96% [88–100%] and a negative predictive value (NPV) of 90% [79–96%]. The positive predictive value (PPV) could not be calculated because there was no true positive (TP) loss of VEP in our series.

The NPV of 90% suggests that preserved intraoperative VEPs indicate preserved visual function. Neurological examination was performed in all patients, but they were only confirmed ophthalmologically in 28 of 46 (61%) patients preoperatively and in 25 of 46 (54%) patients postoperatively. While the neurological examination of our patients includes the testing of visual function, it does not warrant the predictive power of intraoperative VEP as has been reported by others on the basis of ophthalmologic examination [2, 13, 19].

### Stable VEP and new postoperative visual deficit

Intraoperative VEP monitoring could not detect new postoperative deficits in the posterior visual pathway in three patients (patients 3, 8 and 15) with parietal or occipital tumors (6 eyes, FN). All three patients developed homonymous hemianopia postoperatively. In two of these patients, the preoperative visual function was intact and one patient had a preoperative homonymous quadrantanopsia. The intraoperative VEP responses in all three patients appeared stable during resection of the tumor and during closing of the dura. VEP monitoring was stopped after dura closure. In all three patients, there was no intracranial hemorrhage at dura closure. In one patient (patient 8), the postoperative MR images suggest that a hemorrhage or an ischemic infarction, which occurred after VEP monitoring, may have damaged the posterior visual pathway. However, we cannot distinguish with certainty whether the postoperative visual deficit was a consequence of the surgical resection or the postoperative intracranial hemorrhage. As another possibility, the sensitivity of VEP monitoring may have been too low to detect a reduction of VEP amplitude intraoperatively. As suggested by a report in the literature, enhanced sensitivity might be achieved by a lower stimulation amplitude combined with the recording of the electroretinography (ERG) ascertaining an efficient light stimulation axis [13]. Since we could not document a true positive (TP) reduction of visual function in our series, we refrain from predicting postoperative visual outcome but we focus on intraoperative VEP changes as a result of surgical maneuvers.

### Limitations of the study

Postoperative improvements of visual acuity and field could not be detected by VEP recordings. In eight eyes of five patients, the postoperative visual function was improved. The intraoperative VEP recordings of all eyes remained unchanged throughout the surgical procedure. The postoperative improvement of the visual impairment (both visual acuity and field) did not occur instantaneously after surgery, but gradually over several days. Similar findings were reported in other studies suggesting that the affected structures need time to regain their integrity [13,16]. Therefore, intraoperative VEP monitoring, which is limited to the duration of surgery, cannot document functional improvement.

In our study, there were nine patients where VEPs were only feasible in the unaffected ophthalmic nerve (Table 1; Patients 2, 11, 16, 17, 23, 24, 31, 34 and 35). However, visual deterioration is most likely to occur on the affected side with some preexisting deficit. Therefore, preoperative ophthalmologic testing is necessary to predict the clinical utility of intraoperative VEPs in individual patients.

We could not identify the origin of VEP loss, which occurred in two cases (patient 32 and 35, FP) permanently and in one case (patient 33) transiently. Without electroretinography, deviation of the light axis during VEP stimulation could not be excluded. The combination of intraoperative VEP monitoring with ERG will ascertain retinal light stimulation and might thereby improve the clinical utility.

Real time monitoring is not yet possible as we need more than 100 seconds to obtain one waveform. This introduces a delay between a possible damage of the visual pathway, VEP loss and the surgeon’s reaction.

## Conclusions

Satisfactory intraoperative VEPs were feasible in all patients except in those with severe visual impairment. During resection of lesions in the visual cortex, VEP monitoring could not detect new major visual field defects due to injury in the posterior visual pathway. Intraoperative VEPs were sensitive enough to detect vascular damage during aneurysm clipping and mechanical manipulation of the anterior visual pathway in an early reversible stage. Preservation of VEPs predicted preserved visual function. Intraoperative VEP monitoring influenced surgical decisions in selected patients and proved to be a useful supplement to the toolbox of intraoperative neurophysiological monitoring.
