# Correction: Clinical Utility and Limitations of Intraoperative Monitoring of Visual Evoked Potentials

**DOI:** 10.1371/journal.pone.0133819

**Published:** 2015-07-20

**Authors:** Yeda Luo, Luca Regli, Oliver Bozinov, Johannes Sarnthein

There are errors in the Anesthesia management section of the Methods. This section should read: Following our standard protocol for neurosurgical interventions, anesthesia was induced with intravenous application of Propofol (1.5–2 mg/kg) and Fentanyl (2–3 μg/kg). The intratracheal intubation was facilitated by Atracurium (0.5 mg/kg). Anesthesia was maintained with Propofol (5–10 mg/kg/h) and Remifentanil (0.1–2 μg/kg/min). Patients were continuously relaxed with Atracurium (0.5 mg/kg/h) unless motor evoked potentials were monitored.
